# Case Report: Modified Thoracoscopic-Assisted Cervical Resection for Retrosternal Goiter

**DOI:** 10.3389/fsurg.2021.695963

**Published:** 2021-06-09

**Authors:** Cédric Nesti, Benny Wohlfarth, Yves M. Borbély, Reto M. Kaderli

**Affiliations:** Department of Visceral Surgery and Medicine, Bern University Hospital, University of Bern, Bern, Switzerland

**Keywords:** retrosternal goiter, thoracoscopic, minimally-invasive surgery, case report, thyroidectomy

## Abstract

**Introduction:** The treatment of choice for retrosternal goiters (RSG) is surgical resection to relieve symptoms and rule out malignancy. Although the majority of RSG can be removed by a cervical approach only, an extracervical approach (e.g., sternotomy, thoracotomy or thoracoscopy) may be required. Herein, we describe a refined thoracoscopic-assisted cervical two-team RSG resection without thoracoscopic mediastinal dissection.

**Technique:** A 57-year-old man presented with a large RSG with posterior mediastinal extension (PME) and extensive peritumoral vascularization. Due to its extension below the aortic arch and its small connection with the right thyroid lobe, a combined cervical and thoracoscopic approach was intended. The endocrine surgery unit performed the cervical mobilization of the right thyroid lobe, while the thoracic surgery unit gently pushed the mediastinal tumor through the thoracic inlet without performing mediastinal dissection. This allowed a safe visualization of the inserting vessels by the endocrine surgery team at the neck, followed by a stepwise division of the vessels and resection of the retrosternal nodule through the cervical access.

**Comment:** The described approach is indicated for RSG with posterior mediastinal extension, anteroposterior dimension smaller than the thoracic inlet and inaccessibility from a cervical approach only. This minimally invasive approach is associated with a faster recovery, decreased morbidity and postoperative pain, shorter hospital stay and better cosmetic results.

## Introduction

There are several definitions for retrosternal goiter (RSG) with a corresponding high variable rate reported in literature, ranging from 2.8 to 48.0% (1). The two most popular definitions are (1) a thyroid with a lower margin below the thoracic inlet and (2) when >50% of the gland is below the thoracic inlet.

RSG are more often symptomatic than cervical goiter (73 vs. 29%) (1). Symptoms are most frequently respiratory and mild (e.g., dyspnea, cough, choking sensation or orthopnea), but may also be severe (e.g., stridor, respiratory distress, superior vena cava syndrome). The risk of malignancy ranges from 18 to 23% (2). Fine needle biopsy is rarely useful in RSG, since it is not representative for the entire gland.

The treatment of choice for RSG is therefore surgical resection, not least to rule out malignancy. The vast majority can be removed by a cervical approach (>95%) (2). Risk factors necessitating an extracervical approach are a cranio-caudal extent below the convexity of the aortic arch and a retrotracheal location (3). In such cases, it is usually combined with sternotomy to resect RSG with anterior mediastinal extension and with thoracotomy or thoracoscopy in RSG with posterior mediastinal extension (PME). The latter approach is described in a few case series, always combined with a thoracic mobilization and dissection of the mediastinal mass (4–6). However, mediastinal surgical procedures are related to characteristic complications, such as pneumonia, atelectasis, pneumothorax, pleural effusion, and innominate vein injuries with subsequent prolongation of the hospitalization (7).

Herein, we describe a refined mediastinal surgical approach with less morbidity for RSG with PME.

## Technique

**Figure 1:**
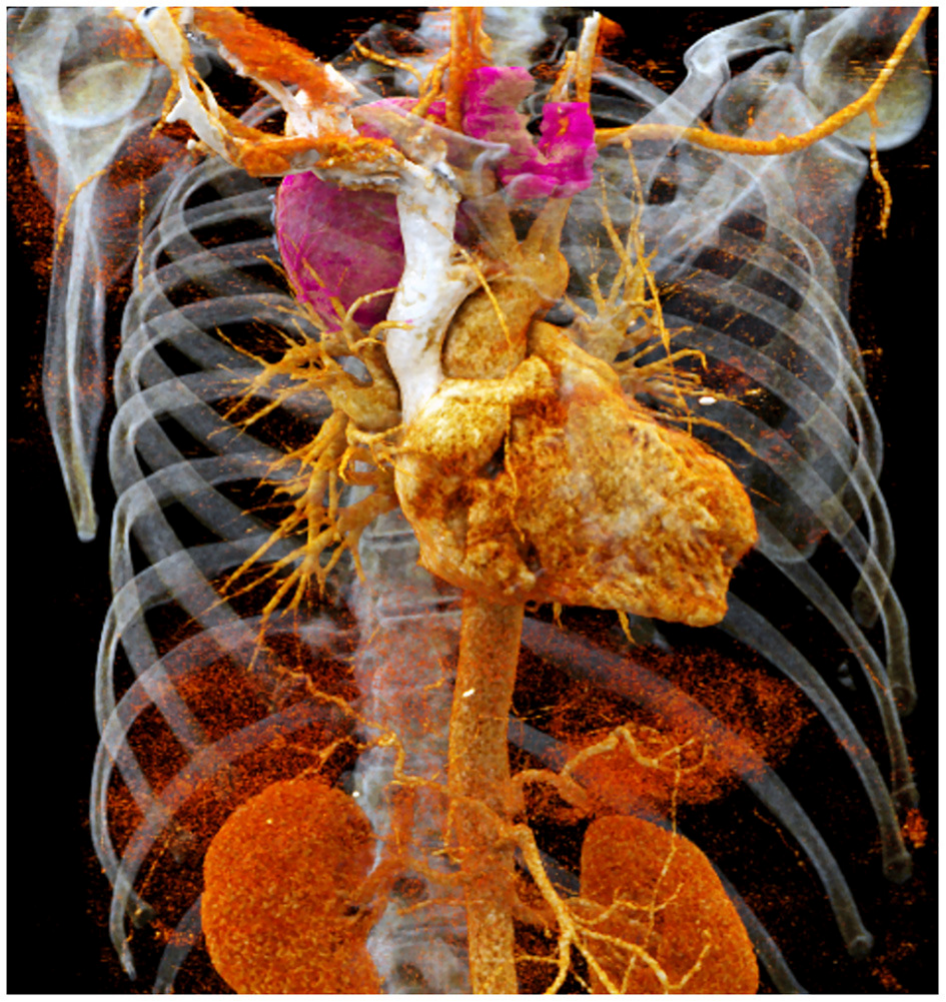
Three-dimensional computed tomography scan reconstruction showing a posterior mediastinal mass, most probably connected with the thyroid by a small tissue bridge and caudally in juxtaposition to the right upper lobe artery.

An euthyroid, asymptomatic and previously healthy 57-year old male patient was referred due to the incidental finding of a posterior mediastinal mass, flanking the right upper lobe artery (Figure 1). Due to a connection to the thyroid by a small tissue bridge, the diagnosis of a RSG with PME was confirmed by iodine-123 scintigraphy. Extensive peritumoral vascularization was visualized by magnetic resonance angiography. Due to the retrotracheal position of the mediastinal mass with an extension below the aortic arch, its small connection with the right thyroid lobe and the extensive peritumoral vascularization, a combined cervical and thoracoscopic approach was intended.

Surgery followed a two-step procedure: The first *part* was performed by the endocrine surgery team. The patient was intubated with a single lumen endotracheal tube and a bronchial blocker for single lung ventilation. He was positioned supine with the neck held in extension. A 4 cm cervical incision was made 2 cm above the suprasternal notch, subplatysmal flaps were elevated and strap muscles dissected. The right thyroid lobe was diffusely enlarged, but not compressing the trachea. Below the sternal notch, the superior edge of the retrosternal goiter was palpable with only a small tissue bridge to the right thyroid lobe. No evidence of vascular supply from the neck was detected. After mobilizing the right thyroid lobe and suture ligating the superior pole vessels, the isthmus was divided.

**Figure 2:**
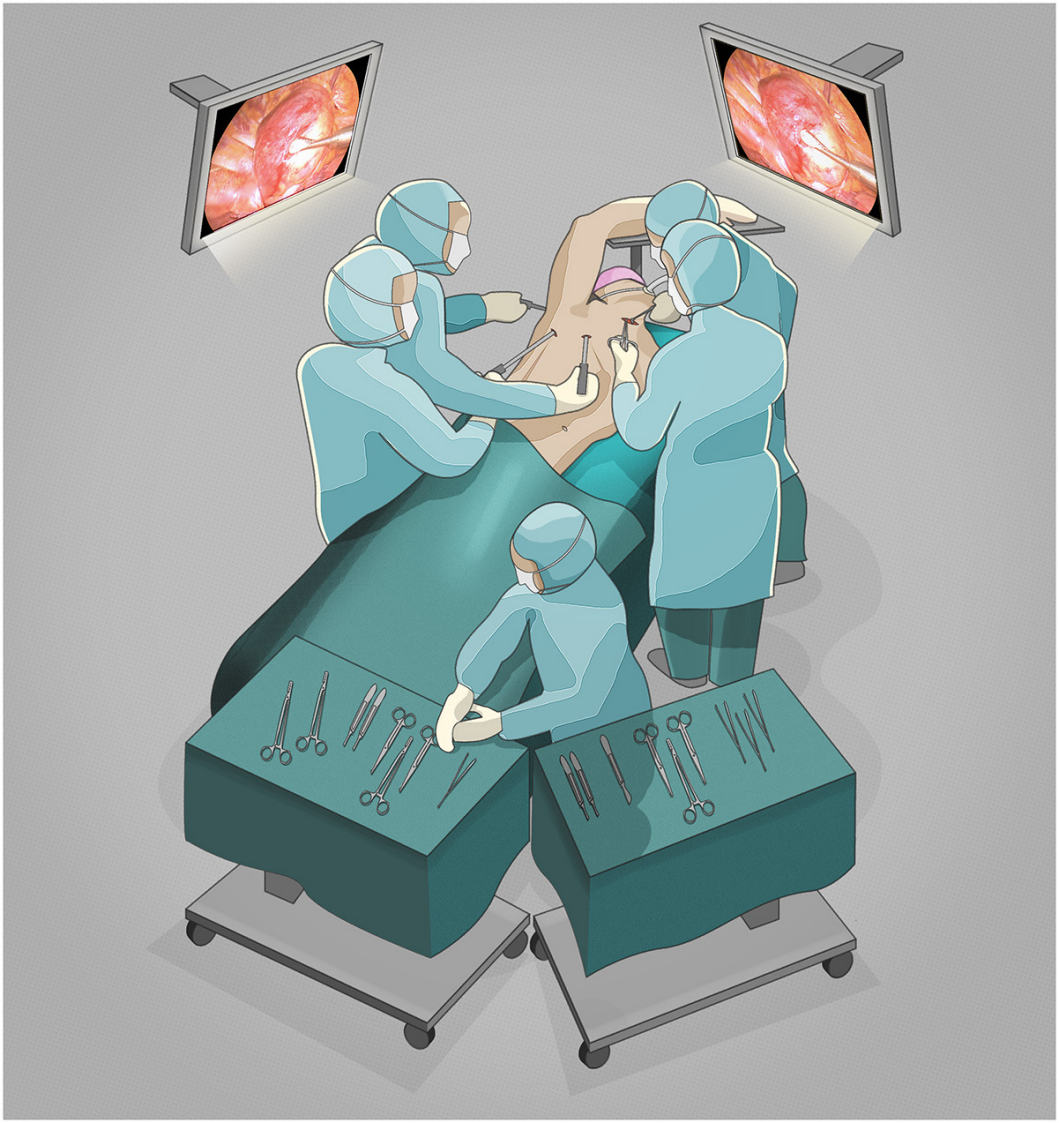
Two-team approach surgical scenario setting.

**Figure 3:**
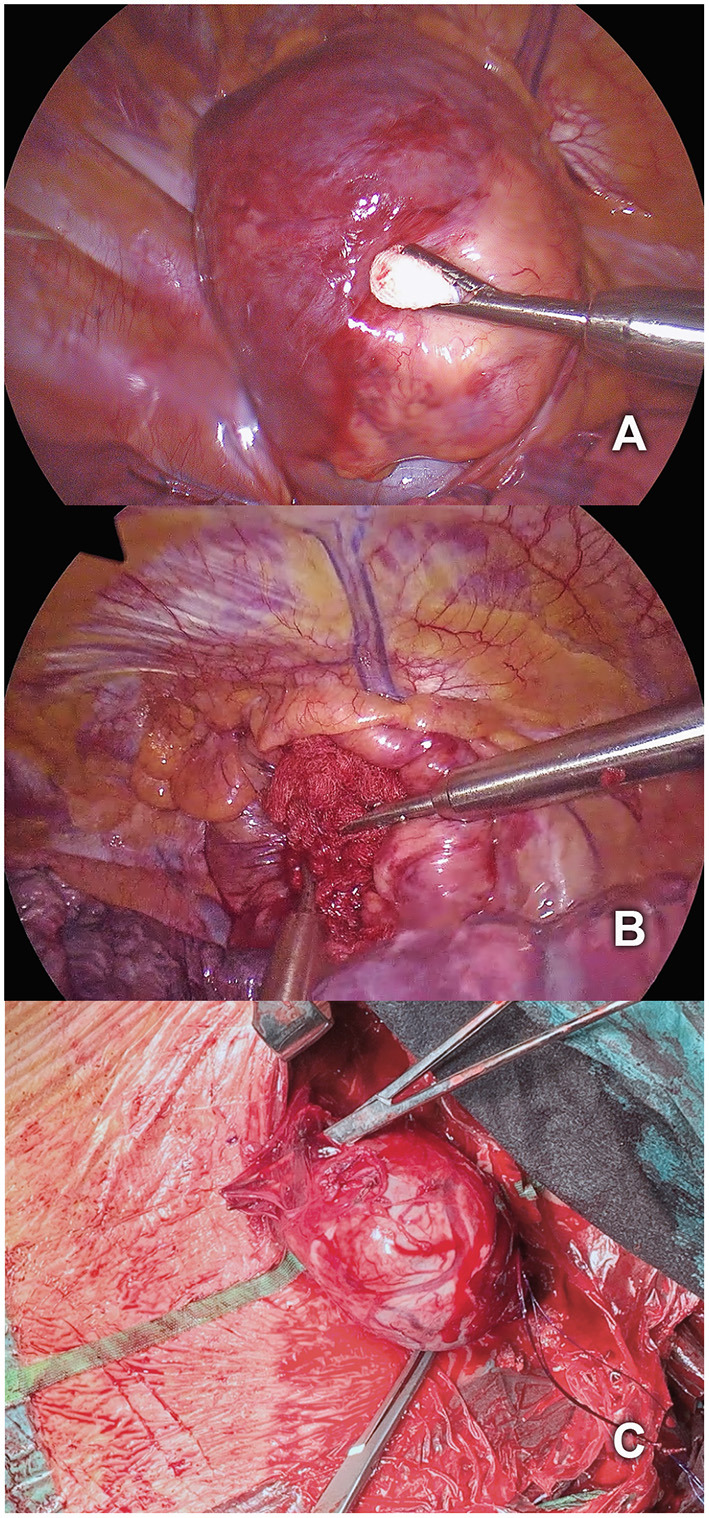
**(A)** Thoracoscopic visualization of the posterior mediastinal goiter. **(B)** Mobilization of the goiter by gently pushing it through the thoracic inlet. **(C)** Simultaneous cervical resection of the right thyroid lobe including the retrosternal nodule.

For the second *part* the patient was placed in the left lateral decubitus position with one-lung ventilation of the left side. Three 10 mm trocars were placed by the thoracic surgery team, standing on the right side of the table (Figure 2): a camera port at the seventh intercostal space in the mid-axillary line, and two additional ports at the eighth intercostal space in the posterior axillary line and at the tip of the scapula with CO_2_ insufflation at 8 mmHg. Thoracoscopy revealed the posterior mediastinal goiter (Figure 3, Supplementary Video 1). The thoracic team gently pushed the mediastinal tumor through the thoracic inlet without performing mediastinal dissection. This allowed a safe visualization of the inserting vessels by the endocrine surgery team at the neck, followed by a stepwise division of the vessels between ligatures. The right thyroid lobe was mobilized in its entirety including the retrosternal nodule and resected through the cervical access. Parathyroid glands were identified and conserved. The function of the vagus – recurrent laryngeal nerve circuit was routinely assessed using the neurostimulator device. A cervical suction drainage was placed into the right thyroid lodge and removed after 24 h.

**Figure 4:**
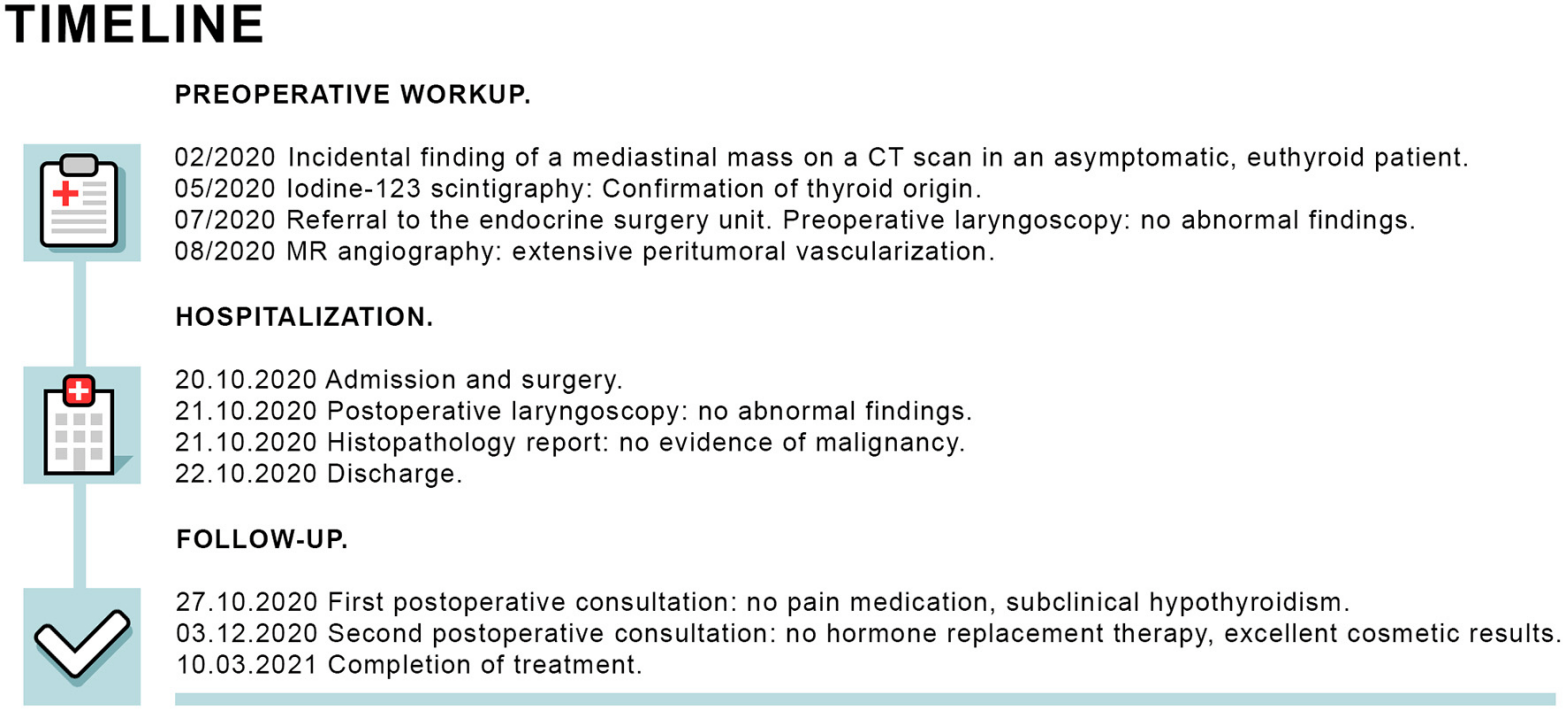
Timeline of preoperative workup, hospitalization and follow-up.

The patient had an uneventful postoperative course with minimal pain and was discharged home on postoperative day two (see timeline in Figure 4).

## Comment

While thoracotomy and thoracoscopy both allow an excellent exposure of the posterior mediastinum, the minimally invasive approach is associated with a faster recovery, decreased morbidity and postoperative pain, shorter hospital stay and better cosmetic results (5, 8). Thoracoscopy, and more recently robotic assisted thoracoscopy, have been used with increasing frequency to approach RSG and are described in a few case series (4–6, 9, 10). However, thoracic mobilization was always accompanied by a dissection of the mediastinal mass. In all patients a chest tube was left in place and postoperative discharge was after 3–7 days (11).

The present approach is indicated for RSG with PME, an anteroposterior dimension smaller than the thoracic inlet, and inaccessibility from a cervical approach only. The pleura remains intact and a chest tube is not needed. As in the present case, extensive peritumoral vascularization can be safely controlled with a stepwise suture ligation of the vessels through the cervical access. Postoperative pain may be reduced and the hospital stay shortened compared to the conventional thoracoscopic approach and similar to an exclusively cervical approach.

## Data Availability Statement

The original contributions presented in the study are included in the article/Supplementary Materials, further inquiries can be directed to the corresponding author/s.

## Ethics Statement

Written informed consent was obtained from the individual(s) for the publication of any potentially identifiable images or data included in this article.

## Author Contributions

All authors listed have made a substantial, direct and intellectual contribution to the work, and approved it for publication.

## Conflict of Interest

The authors declare that the research was conducted in the absence of any commercial or financial relationships that could be construed as a potential conflict of interest.
